# Influence of COVID-19 in the development of delusional ideas disorder. A case report

**DOI:** 10.1192/j.eurpsy.2022.1391

**Published:** 2022-09-01

**Authors:** E. Alvarado Altuve, M. Almodovar Rivera, C. Alcalde-Diosdado Crespi

**Affiliations:** 1 Jaén Hospital Complex, Mental Health Unit, Jaén, Spain; 2 Hospital Complex of Jaén, Mental Health Unit, Jaén, Spain

**Keywords:** social isolation, Covid-19, hypochondria, Delusional disorder

## Abstract

**Introduction:**

Hypochondria is characterized by the presence, for 6 months or more, of a generalized and non-delusional concern with fear of having (or the idea that one has) a serious illness, based on the wrong interpretation of the symptoms. In somatic-type delusional disorder, the delusional idea is fixed, indisputable, and occurs intensely because the patient is fully convinced of the physical nature of the disorder.

**Objectives:**

To describe a clinical case and make a differential diagnosis of hypochondriac disorder vs somatic-type delusional disorder.

**Methods:**

Case report: 61-year-old woman, after suffering from COVID-19, develops a hypochondriacal disorder vs. somatic delusional disorder, presenting anxiety-depressive symptoms and digestive somatic complaints, with a loss of 15 kg of weight. She made frequent visits to doctors and multiple complementary tests discarding organicity. She required involuntary hospital admission for 48 days, and pharmacological treatment with Venlafaxine 150 mg, Olanzapine 5mg, Mirtazapine 30mg and Alprazolam 1mg. The patient presented slow evolution during admission, with ups and downs and stagnation, meriting enteral nutrition due to refusal to ingest, given abdominal kinesthetic hallucinations and digestive evaluation (EDS) with a result of antral gastritis and negative H. pylori. In subsequent follow-ups after partial remission of symptoms, obsessive personality traits are glimpsed, although with better personal functioning.

**Results:**

The diagnosis at discharge was inconclusive, however the data points to a somatic-type delusional disorder.

**Conclusions:**

The influence of COVID-19 as a triggering factor, social isolation and premorbid personality traits, influence the development of a Somatic Delusional Disorder vs Hypochondriac Disorder, regarding this case.

**Disclosure:**

No significant relationships.

